# Key stakeholder perspectives on the barriers and solutions to pharmacy practice towards complementary medicines: an Australian experience

**DOI:** 10.1186/s12906-017-1899-5

**Published:** 2017-08-09

**Authors:** Carolina Oi Lam Ung, Joanna Harnett, Hao Hu

**Affiliations:** 1 State Key Lab of Quality Research in Chinese Medicine, Institute of Chinese Medical Sciences, University of Macau, Room 2057, N22 Research Building, Macao, China; 2 Faulty of Pharmacy, The University of Sydney, A15 - Pharmacy And Bank Building, Sydney, NSW Australia

**Keywords:** Pharmacist, Barriers, Solutions, Pharmacy practice, Complementary medicines, Australia

## Abstract

**Background:**

Although pharmacists are entrusted to play a role in ensuring the safe and appropriate use of all medicines, in general, the inclusion of complementary medicines (CMs) into their professional practice has not been observed. The purpose of this study was to explore the perceptions and opinions of pharmacists and 8 key stakeholder leaders regarding the barriers that hinder pharmacists from providing care related to the use of CMs by patients/consumers and to identify solutions that would support pharmacists’ in extending their role in this area.

**Methods:**

Semi-structured key informant interviews were conducted with 2 practicing pharmacists, 1 pharmacy owner, 1 key representative of a pharmacist professional organization, 1 key representative of a consumer advocacy group, 1 key representative of a medical professional organization, 1 key representative from a complementary medicine practitioner professional organization, 1 leader within a pharmacy school, 2 senior staff from a regulatory authority, and 1 key representative of the complementary medicine industry in Australia.

**Results:**

A total of 9 barriers were identified in this study. Barriers including a lack of CMs knowledge, doubts about the evidence-base, a lack of research skills and access to reliable and reputable information dominated the discussions. A total of 7 solutions were proposed. Of those, the integration of CMs curricula into under-graduate and professional pharmacy education, and defining a clearer role for pharmacists’ standard of practice were considered the most important. Apposing opinions about the role of naturopaths in pharmacies were identified..

**Conclusion:**

It is anticipated that pharmacists will be required to formalise a role in ensuring the safe and appropriate use of complementary medicines to fulfil their professional and ethical responsibilities. However, pharmacists in general are not ready to take up this extended role. Individual key stakeholder groups have considered the existing barriers and have proposed solutions that are isolated measures. To facilitate further developments related to CMs and the professional practice of pharmacy, collaborative efforts between key stakeholders are needed to strategically plan and execute an extended role in a unified manner.

## Background

The use of Traditional Medicine (TM) and Complementary Medicine (CM) is popular in many countries throughout the world including China, United States, European countries, Canada, and Australia [1–9]. Depending on the national regulations, the composition and claims of the products will determine whether they fall under the category of “medicine” or ‘food’. TM or CM products may be referenced differently: “dietary supplements” in the U.S., “natural health products” in Canada, “food supplements” in the United Kingdom, “complementary medicines” in Australia, and “traditional medicine” and “health supplements” in China [9–13]. The terms “traditional medicines (TMs)” and “complementary medicines (CMs)” may refer collectively to the products of Traditional Medicine, Complementary and Alternative Medicine, dietary supplements, herbal medicines, health supplements, vitamins, minerals and natural products.

Studies have shown that TMs and CMs are often used by both the general population and specific patient groups for the maintenance of general health, the prevention of disease and the treatment of minor conditions [14–20]. Throughout the U.S., the U.K., Australia and China, TMs and CMs are accessed via a range of retail outlets such as supermarkets and health food stores. Importantly, a substantial amount of these products are purchased from community pharmacies [7, 21–23]. In the context of consumer autonomy, there is a growing number of individuals who use TMs and CMs as a personal choice in ‘self-management’ and may seek out professional expertise and support during in their decision-making process [24].

Pharmacists have been traditionally charged with the responsibility of ‘gatekeepers’ in ensuring the appropriate and safe use of all medicines [25]. It is argued, that any chemical that is pharmacologically active or has the potential to interact with pharmacotherapy, including TMs and CMs, should fall into the domain of the pharmaceutical care provided by pharmacists [26]. According to the World Health Organization, responsible self-medication with non-prescription products can help reduce the need for medical consultations thereby providing individual and, societal benefits [27]. Good Pharmacy Practice states that pharmacists are expected to maintain and improve their professional performance by taking steps to update their knowledge and skills related to complementary and alternative therapies including TMs and CMs [28]. Pharmacists’ involvement in TMs and CMs has been suggested as a legitimate extension of their established roles in pharmaceutical care [29].

The need for professional guidance in the use of TMs and CMs is a relevant and contemporary issue due to the potential for adverse reactions and TM/CM-drug and TM/CM-disease interactions, especially amongst vulnerable patient groups [30–37]. In support of this, one study reported that the majority of consumers and pharmacists agreed that pharmacists should be knowledgeable about these products, in order to manage drug interactions and to evaluate information that assists consumers in making informed decisions [38]. A recent review suggested that there are 7 major responsibilities related to the pharmacists’ role in ensuring the safe and appropriate use of these products [39]. CMs in particular make a substantial contribution to the business income for many pharmacies [40] further supporting the importance of a role for pharmacists in ensuring their safe and appropriate use.

In Australia, medicinal products containing herbs, vitamins, minerals, nutritional supplements, homoeopathic and certain aromatherapy preparations are referred to as CMs and are governed under the Therapeutic Goods Act 1989 by the Therapeutic Goods Administration (TGA) within the Australian Department of Health [41]. Most of the CMs available in Australia are registered as listed products with TGA and considered safe for sale over-the-counter (OTC) making self-section for self-care easy for the consumer. The regulatory classification of these products as “drugs” make them part of the pharmacist’s professional scope of practice. The use of CMs is prevalent among the Australian population. A 2005 survey indicated that 68.9% of Australians had used a CM product in the previous year [7]. The revenue from CMs exceeded $3.5 billion in 2014, and projections that indicate the revenue for 2017–18 would reach $4.6 billion [42]. The use of CMs is common among the chronically-ill, and CMs are often taken concurrently with conventional treatments [43–54]. Safety issues including adverse reactions and drug interactions have been associated with the use of CMs [55, 56].

Pharmacists in Australia are well positioned to help ensure safe and appropriate use of CMs. Studies have shown that 52.5% of CM users purchased vitamins, minerals and nutritional supplements from pharmacies [23] who attract over half the annual expenditure from this category of CMs [7, 57]. Ethically and professionally, pharmacists assume the overall responsibility when they sell or recommend any medicine including CMs [58–60]. It is proposed by consumers and other health care professionals that pharmacists should be involved in providing information about these products [23, 61–64]. Overall, pharmacists in Australia are quite positive about embracing an extended role in providing information and advising about CMs [15, 41, 65]. However, a gap currently exists between these expectations and pharmacists’ behaviors that are likely to be associated with both known and unknown barriers [66–68, 65, 69, 70].

The objective of this study was to explore the perceptions and opinions of pharmacists and 8 other key stakeholder leaders’ about the barriers that hinder pharmacists in providing care to individuals who use CMs and the potential solutions that would support pharmacists’ in taking up an extended role in this area.

## Methods

### Ethics approval

Eleven semi-structured key stakeholder interviews were conducted with pharmacists and 8 other key stakeholders in Australia in March 2016. The study protocol was approved by the Ethics Committee of the Research Committee at the University of Macao (MYRG2015–00072-ICMS-QRCM) and the Human Research Ethics Committee at the University of Sydney (2016/184).

### Choice of key stakeholders

Based on a systematic literature review, nine key CM and TM stakeholder categories were identified including practicing pharmacists, pharmacy owners, pharmacist professional organizations, consumer advocacy groups, physician professional organizations, CM practitioner professional organizations, pharmacy schools, regulatory authority, and the CM industry [39]. The exploratory research phase involved purposive sampling of key representatives from each key stakeholder category who were approached and invited to participate in an interview. The stakeholder participants all held expertise, leadership, managerial, and/or administrative roles in their respective organizations and were considered to play an influential role in the future direction of TMs and CMs as it relates to pharmacy practice. The participants were chosen strategically in order to optimally elucidate a comprehensive insight from a range of perspectives [71]. A consumer stakeholder leader who also belonged to another stakeholder group was excluded in order to reduce any bias and obtain a true consumer “layman” perspective. Following the purposive sampling stage, a snowballing technique was used to recruit pharmacists known to the pharmacists who participated in the piloting of the interview questions [72].

### Data collection

The pilot explorative interviews were conducted individually with 3 pharmacists. The pilot interviews assisted in refining and targeting the interview questions to be more specific in preparation for the stakeholder interviews, resulting in a complete interview guide. A participant information statement outlining the study was provided to all the participants prior to the start of the interview and signed consent was obtained. The interview questions were developed to answer the study objectives. All interviews were recorded and managed in accordance with the approved protocol.

Participants who held multiple roles, either in practice and/or by profession, were encouraged to provide opinions from their different perspectives. All stakeholder representatives who were invited to participate in this study participated.

A total of 11 interviews were conducted with each lasting for 45–60 min. All interviews were conducted in English. Nine of the interviews were conducted face to face and audio-recorded with formal consent from the participants. The other 2 interviews were conducted via telecommunication and were audio-recorded with verbal consent. Interviews continued until saturation was reached for the key emergent themes.

### Data analysis

On reaching theme saturation, the interviews were transcribed verbatim. Two investigators participated in establishing preliminary coding categories, and later coded all transcripts individually. Meetings within the research group were held regularly to come to consensus on any areas of disagreement. Qualitative techniques were employed to identify themes, general trends, major issues, differences, and unique individual responses. These findings were manually identified from the transcripts by two investigators to ensure accuracy of interpretation and contextualization of the participants’ comments. Content analysis was used to identify specific barriers and solutions with regards to community pharmacists’ role related to CMs.

## Results

A total of 11 key stakeholder interviews were conducted with 2 practicing pharmacists, 1 pharmacy owner, 1 key representative of a pharmacist professional organization, 1 key representative of a consumer advocacy group, 1 key representative of a physician professional organization, 1 key representative from a CM practitioner professional organization, 1 leader within a pharmacy school, 2 senior staff from the regulatory authority and 1 key representative of the CM industry. All the participants agreed that pharmacists should assume responsibility for the CMs they sell or recommend. However, the participants believed that in general, the care provided by pharmacists related to CMs was substandard. In light of this situation, participants provided insights about the barriers for pharmacists providing quality care related to CMs use and proposed a number of solutions.

### Barriers hindering pharmacists’ duty of care regarding CMs

Table 1 summarizes the 9 barriers identified by the participants. Insufficient knowledge about CMs was suggested by all participants as a fundamental barrier. Four other barriers were discussed by the majority of the participants: pharmacists’ attitude towards CMs (8 participants); lack of research skills (7 participants); lack of evidence for efficacy and safety of CM (6 participants); and lack of access to trustworthy information and support (9 participants). Lack of time (2 participants), consumers’ attitudes (4 participants), lack of a defined role for pharmacists (2 participants) and poor inter-professional communication with doctors (2 participants) were also seen as barriers by less than half of the participants.

1. *Insufficient knowledge about CMs (all 11 participants)*

**Table 1:** Barriers hindering pharmacists from providing care for patients/consumers using CMs

Barriers		Practicing Pharmacist 1	Practicing Pharmacist 2	Pharmacy owner	Pharmacist profession al organization	Consumer advocacy group	Doctor professional organization	CM practitione r profession al organization	Leader within a pharmacy school	Regulatory authority senior staff 1	Regulatory authority senior staff 2	CM industry
1. Insufficient knowledge about		YES	YES	YES	YES	YES	YES	YES	YES	YES	YES	YES
2. Pharmacists’attitude	Being skeptical									YES		YES
	Lack of initiative	YES		YES	YES				YES		YES	
	Lack of confidence and communication skills	YES				YES						YES
3. Lack of research skills		YES			YES	YES			YES	YES	YES	YES
4. Lack of evidence for efficacy and safety of CM s		YES			YES	YES			YES	YES	YES	
5. Lack of access to reliable and reputable information and support		YES	YES	YES		YES	YES	YES		YES	YES	YES
6. Lack of time				YES		YES						
7. Consumers’attitude	Misperception about CMs				YES				YES			
	Pharmacists don’t know much										YES	YES
8. Lack of defined role					YES				YES			
9. Miscommunication with doctors					YES				YES			

The general opinion was that pharmacists’ ‘sub-optimal’ practice behaviors related to CMs was strongly associated with a lack of knowledge about CMs. Apart from some new graduates who may have been exposed to CMs in their undergraduate training, the majority of pharmacists were not trained to provide CMs information or counselling on the efficacy or safety. It was indicated that:

*“Education and information resources are the biggest gap right now and vulnerably open, not quite equipped when it comes to these issues. It is very hard for pharmacists to have all the knowledge given it is such a large range of products. The sources of data is also a bit patchy. There is a lot of new products, market driven. To keep current is also a challenge.”* (*Key representative of a consumer advocacy group)*

The lack of knowledge was attributed to a deficit in the undergraduate curriculum as the importance of CMs was not generally recognized at a university level. It was mentioned that:

*“I think lack of knowledge very much come from the deficit in the undergraduate curriculum and I think the reason for that deficit is because you find that some people who head up the pharmacy faculties don’t see that is important or maybe have a bias against it.”(Key representative of the CM industry)*

According to another interviewee, pharmacists had been ‘calling out’ for educational materials on CMs as part of the pharmacy training for nearly two decades. However, this request had not been responded to effectively. It was indicated that:

*“We did an educational need analysis primarily for our own benefits. Pharmacists at that stage were crying out for graduate education in complementary medicines. That was in the early 2000. Everything that I have seen where they have done this sort of educational need analysis since, they are coming up with the same sort of findings. Basically, pharmacists are in the situation where they need to start to demand their professional and educational bodies provide appropriate education.” (Key representative from a doctor professional organization)*

2) *Pharmacists’ attitudes towards CMs (8 participants)*

Despite previous studies reporting that in general pharmacists are positive about their role in CMs, the findings of this study suggested otherwise. Most of the participants perceived that pharmacists have a negative attitude related to the lack of initiative (5 participants), a lack of confidence and communication skills (3 participants), and are skeptical (2 participants),

According to 5 participants, CMs were often considered secondary and more of a retail product by many pharmacists. Two participants argued that there was already too much for pharmacists to know and keep up to date within the area of pharmaceutics and it would be unreasonable to ask pharmacists to take up any additional roles. Pharmaceutics were ‘considered riskier’ and they were the core to the profession and the business. Moreover, as CMs could be obtained through other retail outlets, pharmacists would hesitate to assume the ultimate responsibility of ensuring the appropriate and safe use of CMs. Some participants believed that pharmacists were aware of their duties but interestingly they did not think it related to CMs. It was indicated that:

*“I think there is a mismatch between the growing pharmacy business which has been growing at an exponential rate with complementary medicines but that has not been reflected in the knowledge base increase exponentially for pharmacist or the consent that I actually making so much money in complementary medicine so I should know more about and giving back more advice. There is no correlation or probably inverse correlation. It is quite discouraging. Pharmacists are aware of their duties but somehow they don’t think it relates to complementary medicines.”(Leader within a pharmacy school)*

*“They probably do have different level of professional service and different attitude. Because probably many of them don’t have the knowledge. The other thing is that in their minds, they don’t think complementary medicines carry big risks to become a concern.” (Senior staff from the regulatory authority)*

Three participants were concerned that pharmacy graduates lacked the confidence to have a conversation with patients/consumers about pharmaceutics and other OTC products let alone CMs. The qualities of self-motivation, confidence and communication skills were suggested to be lacking among young pharmacists. Pharmacists’ lack of motivation to learn about CMs and the evidence for efficacy was considered another contributing factor to a reactive rather than proactive approach in this area. It was indicated that:

*“The other issue is confidence. I mean you would find a lot of pharmacy graduates, even if they know a lot about OTC, they lack of the confidence to have the conversation. I also think that the way pharmacy students gain entry to the course need to be looked at in general to make sure that you have people with good communication skills regardless if it is about complementary medicines or even prescription medicine and OTC, and that they do come out from behind the counter and talk to people. It is a lack of motivation and lack of confidence.”(Key representative of the CM industry)*

*“There will be a small number that will talk to a pharmacist. The flip side of that very often pharmacist don’t come out and talk about these because they don’t have the training or the confidence. In an ideal world, pharmacist should be actively involved in that discussion. The reality at the moment is that they are not.”* (*Key representative of the CM industry*)

*“My experience when I go to pharmacy to buy complementary medicines, I don’t go ask the pharmacist. I don’t even see one on the floor.”* (*Senior staff from the regulatory authority*)

Pharmacists could be excessively skeptical about the safety and efficacy of these products as suggested considered by 2 participants. It was indicated that:

*“The major barrier would be the people like me who are skeptical. I am not an advocate of complementary medicines mainly because I am a scientist.” (Senior staff from the regulatory authority)*

*“The biggest barrier in health care is that doctors and pharmacists won’t learn the evidence. I am questioning whether pharmacists know what evidence means quite honestly. They think they know and then they would turn it around like a shield of evidence-base, but what kind of evidence are you talking about? What is evidence? I think they don’t even have that understanding.” (Key representative of the CM industry)*

Some participants raised an important point that pharmacists who did not ‘believe’ in CMs, yet chose to sell them without providing any professional service or advice face a professional dilemma.

3) *Lack of research skills (7 participants)*

The perception of most participants was that pharmacists in general, were not equipped with the appropriate skills to identify quality research or critically interpret the results. Analytical skills were considered very important by most participants for interpreting the available evidence about the safety and efficacy of CMs. One participant even doubted if pharmacists would really know what evidence actually meant and if they were able to understand scientific evidence. Another participant suggested that evidence based medicine had been used by some pharmacists as an excuse to avoid any conversation about these products. It was indicated that:

*“Anyone can go on the PubMed but I think pharmacists are not very well trained to read the scientific papers. Even when you give them the hard-core scientific evidence, a lot of them did not really understand it. It is fascinating.” (Key representative of the CM industry)*

*“It would also be hard for some pharmacists to interpret a good study from a not so good study even if they log on a database. How well trained they are in that. It is going to take them time to pick a good quality study.” (Key representative of a consumer advocacy group)*

*“I mean not only the knowledge base but also being able to filter and assess in an independent way. People choose what they believe or what not to believe but pharmacists should be able to filter through the information and sort the garbage through.” (Leader within a pharmacy school)*

*“Very often pharmacists don’t interpret what is available to them is information the same way I would with the same scientific thinking and clinical capabilities.” (Key representative of a pharmacist professional organization)*

*“Pharmacists need the skills and experiences like people who have done research to look through the literature to interpret the findings. This is a skill to learn from doing research. But general pharmacist may not have the time and skills.” (Senior staff from the regulatory authority)*

4) *Lack of evidence for efficacy and safety of CM (6 participants)*

According to the regulatory system for CMs in Australia, the majority of CMs were listed products which meant they were subject to stringent evaluation by the regulatory authority for quality and safety but not efficacy. While most participants believed that some evidence existed to support some health benefits of some CMs, the efficacy profile was usually not complete. Without a full demonstration and understanding of the efficacy of CMs, participants agreed that it would be difficult for pharmacists to provide information about these products in a responsible manner. With regards to the safety issues, it might not necessarily be about individual single-ingredient CMs. Rather, the challenges were to predict the safety of multi-ingredient formulations taken concomitantly with pharmaceutics. It was indicated that:

*“The main drawback in Australia and the main drawback all over the world is that there is no proof of efficacy when you register complementary medicines to the best of my knowledge. That is the problem. What is happening is that the practitioner is standing on a ground that is moving. And this is unfair. Grossly unfair. So that makes it doubly difficult to people. It goes back to the proof of efficacy.” (Practicing pharmacist)*

*“That is the safety of a product that I see as a major barrier. Again, because we don’t know what is in there. It is getting probably better in Australia. But then again, even if we know what is in there, the studies hasn’t been done to prove that they are safe and of course efficacy we don’t know yet. The science and evidence is the main barrier.” (Senior staff from the regulatory authority)*

It was proposed, that the CM industry are not adequately incentivized to conduct original research for 2 reasons: (1) The industry realized that the availability of quality evidence to support efficacy had, if any, minimal correlation with the volume of sales since neither the consumers nor the pharmacists, as a general rule, assessed the quality research to support a CM products use; (2) No intellectual protection measures were in place to prevent the use of results by competitors in the development of equivalent products. It was indicated that

*“If I were to fund half a million for a clinical trial on a particular ingredient, every other company can copy that. I think that is a failure from the regulator who is actually not promoting innovation and advance in sciences.” (Key representative of the CM industry)*

5) *Lack of access to trustworthy information and support (9 participants)*

A general concern arising from the 11 interviews related to a lack of trustworthy information and resources about CMs. Four participants said that they did not know of any reliable information about CMs that were readily available to pharmacists. However, three other participants knew that some CMs were included in key easily accessible resources such as the Therapeutic Guidelines, Australian Medicine Handbook and The Australian Pharmaceutical Formulary. One participant pointed out that on the list of CMs references recommended by the Pharmaceutical Society of Australia, there were two evidence-based CMs references. Other reliable information might come from continuing professional development/education articles, journal articles and professional newsletters. However, one participant pointed out that at this stage, pharmacists needed to be proactive in accessing such information despite it sometimes being difficult to access and costly. It was indicated that:

*“I think it will be better to have more details about quality control, about safety and about existing data available on efficacy. Ideally, even with some data about interactions. Those information should be available for public. The more data or quality, validated information available, the better for the public and for the industry. I will say at this stage, all this information is quite limited and we need more information.” (Key representative from a CM practitioner professional organization)*

*“There are a number of resources available to them (pharmacists) but a lot of them are not aware of them. For example, they might rely on the Australian Medicine Handbook or Australian Therapeutic Guidelines. They will look at those and think there is not much complementary medicine information in there if any. And they would assume that there is all there is. They didn’t realize that there are many other dedicated databases and text books specifically focusing on these areas.”(Key representative of the CM industry)*

*“The resource of reliable information is not readily to pharmacist. You have to make a special effort.” (Practicing pharmacist)*

*“Right now pharmacists rely on to large degree information from product sponsors. So products come in, they get training and information. I think they rely on and read what sponsors provide. Hopefully, this information is reliable and reputable. But things are not certainly arm’s length. I am imaging that they could be just like consumers and google about things. But it is a key challenge to have a single source of credible information unbranded.” (Key representative of a consumer advocacy group)*

6) *Lack of time (2 participants)*

Some participants argued that pharmacists were already very busy with pharmaceutics. As explained by one participant, given such a huge range of CMs and new products keep ‘pumping’ into a market-driven environment, it is hard for pharmacists to be up-to-date. It was indicated that

*“I think it is too much to ask for pharmacists to take up more roles when they sell CMs because there are already so many medicines, and there is already too much for a pharmacist to know. Mentally, I even found it so challenging to know all these medicines, all that contraindications, how they work and there is just so much polypharmacy and all the new drugs always coming in and you have to keep CPE. Those things are riskier and they are core business. They are the things that carry more health risks if they are wrong so I think we have to concentrate on those. CM are lower risks and are secondary to me.” (Practicing pharmacist)*

7) *Consumers’ attitudes towards CMs (4 participants)*

Two participants suggested that consumers’ attitudes also impacted the pharmacists’ role related to CMs. To many consumers, their major source of information about CMs is from advertising materials and from word of mouth through their family and friends. Collectively, their assumptions about the safety and efficacy of CMs, a trust in the regulatory authority and the CM industry and pharmacies, consumers could easily perceive or rather misconceive that all CMs are safe. They would then no longer see the need to make enquiries at the pharmacy. Trying to manage the consumer’s belief system was considered a significant challenge. On the other hand, some consumers might have experienced that pharmacists were not able to provide the CMs information they needed and therefore decided not to ask pharmacists about CMs again. It was indicated that

*“The biggest challenges would be ….. trying to manage consumer’s misperception about the implied endorsement of safety and efficacy of a product being sold in the pharmacy.” (Key representative of a pharmacist professional organization)*

*“The reality is also that people don’t actually ask pharmacist because they know pharmacists don’t know the answer. Unfortunately, pharmacists have proven it that they don’t know the answer. So there is a lot of work to be done on both sides.” (Key representative of the CM industry)*

8) *Lack of a defined role for pharmacists (2 participants)*

As one of the participants mentioned, a lack of a defined role in CMs implied unclear responsibilities and the potential consequences arising from this ambiguity. Two participants did not make an association between the pharmacist and CMs. It was indicated that:

*“There is no absolute legal requirement at the moment. At the moment, pharmacists can recommend it freely without worrying about it. If you take it to a higher policy level, the National Medicine Policy that was put out in 2014 stated that all roles and responsibilities in the quality use of medicines and the definition of medicine included Prescription, complementary medicines and OTC so you can take it that even though we may not pin dart on a dart board on complementary medicine, it is part of overall provision of medicine.” (Leader within a pharmacy school)*

*“There is certainly not an agreement on how this can be managed. Whether they constitute the same degree of care as pharmaceutical prescription medicine. It is generally more polarized rather than balanced from this viewpoint.” (Leader within a pharmacy school)*

*“We have a law requiring us to have people supervised when we talk about medicines, but there is no supervision required with complementary medicines.” (Key representative of a pharmacist professional organization)*

9) *Poor inter-professional communication with doctors (2 participants)*

Two participants suggested that many doctors are uncertain about the safety and efficacy of CMs and strongly advise against the use of these products to their patients due to the potential risks of delaying or interference with conventional treatment. Disapproval by doctors of a patients’ CMs use was also one of the major reasons for patients’ not disclosing their use of CMs. For pharmacists selling or recommending CMs without evidence is seen as a contradiction to their professional role. This perception could easily discourage pharmacists’ motivation to establish communication with doctors about the patients’ use of CMs. It was indicated that:

*“The doctor said to me, ‘If you have been taking it seriously as a health care professionals, you could have got rid of all the shit out at the front. All the bloody vitamins and stuff.’ The fact that we are selling them, talking about them or even promoting them will be enough to make my GP upset about them.” (Key representative of a pharmacist professional organization)*

*“You could argue that you want to make sure the doctors know they are taking these things (complementary medicines). There is a privacy issue though. So you don’t want to ring up the doctor and tell him your patient took this from your pharmacy.” (Leader within a pharmacy school)*

### Solutions to support pharmacists’ extended role in these products

As presented in Table 2, a total of 7 solutions were suggested by the participants. The integration of CMs into pharmacists’ undergraduate and professional education development was discussed by all participants. A clear definition of the pharmacists’ role in CMs (5 participants), pharmacies employing a naturopath (8 participants), the establishment of reliable, easily-accessed information (10 participants), promoting quality CMs research (2 participants), collaboration among health care professionals (2 participants) and the provision of consumer education (2 participants) were also considered important to support the pharmacists’ extended role in CMs.

1. *Integration of CMs into pharmacists’ education (11 participants)*

**Table 2:** Solutions which support pharmacists’ extended role in the area of CMs

Solutions		Practicing Pharmacist 1	Practicing Pharmacist 2	Pharmacy owner	Pharmacist professional organization	Consumer advocacy group	Doctor professional organization	CM practitioner professional organizatio	Leader within a pharmacy school	Regulatory authority senior staff 1	Regulatory authority senior staff 2	CM industry
1. Integration of CMs into pharmacists’ education	University training	YES	YES	YES	YES	YES	YES	YES	YES	YES	YES	YES
	Internship training	YES										
	CPE	YES	YES		YES		YES	YES		YES	YES	YES
	Post graduate training						YES	YES		YES		YES
2. Clear definition of the pharmacists’role in CMs					YES	YES	YES	YES				YES
3. Pharmacies employing a naturopath	YES				YES	YES	YES	YES		YES	YES	YES
4. Establishment of reliable, easilyaccessed information	Database			YES	YES	YES			YES			
	Product information endorsed by authority					YES				YES	YES	
	Others validated information							YES			YES	YES
	Guidelines/Red flag system	YES	YES									
5. Promoting quality CMs research			YES			YES						
6. Collaboration among health care professionals						YES			YES			
7. Provision of consumer education									YES			YES

Education was considered central to improving pharmacist’s practice related to CMs. As enquires about these products is becoming an increasingly significant part of the daily routine, pharmacists needed to be proactive in obtaining CM education to improve knowledge and confidence. As one participant explained, being equipped through education and training had both professional and economic benefits. Participants suggested that some consumers would be more likely to seek professional guidance and purchase CMs if a pharmacist could demonstrate they had a good understanding of CMs.. Unless pharmacists were knowledgeable about these products and maintained their professionalism, they are potentially jeopardizing not only consumers’ health but reducing customer satisfaction. Arguably, pharmacists should be incentivized to attain CM education which could be delivered in undergraduate degree programs, internship training, continuing professional development (CPE) and post graduate training. It was indicated that:

*“They also need to receive some training in herbal medicine or complementary medicines in order to enable them to handle them because they are under different philosophy and different framework with different clinical applications.”(Key representative from a CM practitioner professional organization)*

*“Unless pharmacists try to get the same level of education about complementary medicines themselves, they will be deficient and they will feel insecure and they will feel unhappy, unsettled, because the complementary medicine requests and complementary medicine information are probably requested several times every day. So that is the professional and economic reality of it.”(Practicing pharmacist)*

All participants emphasized the importance of integrating CMs into undergraduate pharmacy education in order to ‘sensitize’ pharmacy students to these products. This was believed to be critical to make sure pharmacists would communicate about these products the same way as they would any other medicine. A concern was raised that the current CPE requirements allow pharmacists to pick and choose CPE topics based on their personal interests. This could result in pharmacists limiting or receiving no training about CMs. It was indicated that:

*“I think something we are trying to do here is to make sure they treat them the same way as pharmaceutical medicine in terms of to the approach to the conversation with the patients. So it is managed from a communication viewpoint in the same way.” (Leader within a pharmacy school)*

*“The inclusion of more content into the undergraduate curriculum is very critical. Because you will find a lot of pharmacy graduate once they leave the university, when it comes to CPE and ongoing education, they can pick and choose. So if they don’t have any interests in this area, they can then keep choosing to attend those kind of lectures that are in different areas then they are going to completely miss the education.”(Key representative of the CM industry)*

*“I think it is important for the current education to ensure university graduates of doctors and pharmacists to have more information and knowledge. Complementary medicines only took off in the last 10 to 20 years. So people should start to learn more.”* (*Senior staff from the regulatory authority)*

Some participants suggested that at the very least, the undergraduate curriculum should cover the top-selling CMs equipping graduates with evidence-based knowledge regarding the efficacy and safety of common CMs. It was indicated that:

*“I think I would appreciate it if at least they taught about the top 20 baseline stuff. Because a lot of that stuff I would have had to learn on my own. And that’s only because I have had an interest in it.” (Pharmacy owner)*

Another important aspect of undergraduate training identified in this study was research and analytical skills. As one participant illustrated, knowledge always changed and what pharmacy students learnt in the university might change over time. Therefore, the ability to independently filter, interpret and select accurate information was critical when trying to provide advice to people. The volume of information and published literature is overwhelming and could sometimes be misleading. It was suggested that pharmacists needed the skills and experience to search the literature, to interpret the findings, to determine the quality of the clinical studies and to regularly revisit the literature to ensure currency.

One participant was particularly concerned about pharmacists’ internship and mentioned that continuous training of CMs should be emphasized in the internship training as this was the time when pharmacy graduates had an opportunity to experience the issues related to CMs in day-to-day practice. The aim of internship was for the pharmacy graduate to apply their knowledge and skills in CMs in the practice setting. It was indicated that:

*“If it (complementary medicine education) is not happening in university, then it should be in their internship year. Because what they do is in that 12 months, they are going to be in the pharmacy that has the issue happening then the aim of the exercise is they will be able to use university skills to interpret or read or know that they must find out. To my way of thinking, that is when pharmacists start to gain some knowledge and perspective about complementary medicines that are important to them.”(Practicing pharmacist)*

For the pharmacists who had already registered, continuing professional education (CPE) was one of the major ways to keep up-to-date with new findings about the safety and efficacy of CMs. Most participants agreed that if pharmacists were working with patients who used CMs, they should include training related to CMs in their CPE plan. As suggested by 4 participants, taking into consideration the costs and time related to undertaking CPE, a structured CPE plan that included a compulsory CMs element could be useful. Other incentives should also be in place to help pharmacists maintain interests in seeking training in this area. It was indicated that:

*“Any professional requirements that requires additional training means you are taking people out of their day-today duties. This also means that they will lose income and they need to pay money to learn new skills. Unless things have become more mandatory or people will choose those things, it will be less likely to be able to achieve high level of compliance. The barriers will be like additional training, costs, taking them out of the job. The problem or the question is that will additional qualification allow tem a higher level of income. For example, if you are able to handle more than just western medicines, and also complementary medicines, will you get a higher level of pay rate or a higher level of income to reward additional learning?” (Key representative from a CM practitioner professional organization)*

If pharmacists were to provide information and clinical advice, they should have formal training comparable to a post-graduate level. Based on an interviewee’s experience, pharmacists with additional qualifications in naturopathy or CM are in a better position to guide consumers in making informed decisions.

2) *A clear definition of the pharmacists’ role in CMs (5 participants)*

Five participants believed that defining their role in CMs more clearly would make a substantial difference in pharmacists’ standard of practice. In addition, this definition would be pivotal in developing and implementing the necessary educational programs and practice guidelines for pharmacists to fulfil such a role. It was indicated that:

*“I think they need to identify the role of pharmacist in handling the complementary medicines. It needs to clearly define the role whether pharmacists are just selling the products or if they are providing health advice. And then from there, it is to design a very clear articulated capability requirements for these people to be adequately plays these roles and from there you can identify key knowledge, skills and attributes required. This will lead to the final design of the training course o enable the people to achieve the outcomes.”(Key representative from a CM practitioner professional organization)*

*“If we don’t have a policy about these things, there is no trust in our professional generally. I think what will happen over time is that the statement (The Complementary Medicines Position Statement by the Pharmaceutical Society of Australia) will get tighter and the only way for it to get tighter is to provide more examples and clinical example into it.”(Key representative of a pharmacist professional organization)*

Three participants had doubts whether it was appropriate for pharmacists to take on a role related to CMs. It was indicated that

*“Should complementary medicines be highlighted as part of pharmacists’ role? It is a complex question. There is a general role for pharmacist. It is unfair at this point of time given registered pharmacists have not received undergraduate training in complementary medicines to be developing role for them that is largely being a role for naturopath and other TM practitioner where all of a sudden we are saying that you should be having responsibility in this area too because you are selling them.” (Key representative of a consumer advocacy group)*

*“I agree that it is quite unfair to ask pharmacist to really provide certain level of service with Complementary medicines as they are not trained. I think they should be trained before handling the products because herbal medicine are not naturally all safe.”*(*Key representative from a CM practitioner professional organization)*

*“If you read the actual code of ethics of pharmacists, it basically says that pharmacists are required to be aware of the underlying pharmacology of all the things that are actually dispensed in the pharmacy. I don’t think anybody would want it to be explicit. Because if it were made explicit at the moment, virtually every pharmacist in the country would be in breach of that ethical code.”*(*Key representative from a doctor professional organization)*

3) *Pharmacies employing a naturopath (8 participants)*

According to one participant, 1 in 5 pharmacies in Australia employ a naturopath and two-thirds of consumers would be very happy for pharmacies to have a naturopath in the store to provide CMs information. The responses to this strategy were mixed. On the positive side, as part of a standard duty of care, if a pharmacy supplies CMs, there should be at least one staff member qualified to handle related enquiries. To have a naturopath in the pharmacy meant the pharmacists were trying to provide or facilitate the best care that they could. It was indicated that:

*“With the research that I do, we have actually showed that two-thirds of people would be very happy for pharmacies to even have naturopaths in the store so they will have someone who is an expert to talk about these because they are recognizing that pharmacists don’t know enough.” (Key representative of the CM industry)*

*“If you have a nutritionist or a naturopath, these people will be the expert on nutrition and complementary medicines and pharmacist can work in collaboration.” (Key representative of a consumer advocacy group)*

*“Ideally, it should be the pharmacist who should deal with consumers’ complementary medicine use. But in the interim of what we have, I am happy to have a naturopath in my practice to deal with the situation. Having a naturopath in the pharmacy is an attraction to the customers.”(Key representative of a pharmacist professional organization)*

*“I think pharmacists basically need to actually either know the medicine that they actually have in the pharmacy or they need to actually have a content expert on hand to be able to discuss the questions.”(Key representative from a doctor professional organization)*

However, there were two interrelated limitations to this proposed solution. In Australia, Naturopathic practitioners are not governed by statuary regulation and this is associated with significant variations in education. The implications for pharmacists who employ naturopaths is that they are ultimately responsible for the advice given by the naturopaths. Other ethical and legal implications need to be considered when a statutory regulated profession takes advice from a self-regulated profession such as naturopathy or herbalism. The implications for the consumer relates to a potential lack of standardized naturopathic advice across pharmacies that may lead to confusing or unreasonable expectations and misplaced trust. It was indicated that

*“The pharmacist is the captain of the ship. The naturopath, even though they are independent practitioner, they are like pharmacy assistant to pharmacist in a pharmacy. They are assisting pharmacist. As a pharmacy assistant, they need to make sure what they are doing is acceptable to pharmacist. So if the pharmacy assistant is recommending a product, then the pharmacist must know what is happening in the end.”* (*Practicing pharmacist)*

*“One trend that has been occurring in Australia pharmacies is a lot of pharmacists are employing graduated naturopaths. A difficulty is that the naturopathic medicine is not yet a registered health profession in this country and so the interested still places the liability on the actual pharmacists and that creates some dilemma. There are some risks associated with that is that they are using a non-regulated professional at the moment.” (Key representative from a doctor professional organization)*

4) *Establishment of reliable, easily-accessed information (10 participants)*

Most participants agreed they need access to reliable and reputable resources to assist pharmacists in providing information and/or advice about CMs. Details about quality control, safety, and current efficacy and safety data including interactions are required for both the pharmacists and public to make informed decisions about CMs use. A well-managed database was the preferred source for CMs information as hardcopies were considered to date quickly. As pointed out by one participant, development and maintenance of such databases was a massive and long-term commitment that faced challenges such as allocating the right body and attracting sufficient funding. From the regulatory standpoint, 2 participants suggested that the TGA could review the regulatory requirements of the product information in and on the packaging of CMs to include pre-assessed important information for easy reference by pharmacists and consumers. This would help pharmacists and consumers gain a better understanding about the indications for use, precautions and potential drug-drug interactions. Practice protocols could also be developed to facilitate good practice when pharmacists were providing information and counseling consumers about CMs and in conducting of medication reviews. It was indicated that:

*“There should be a red-flag system to help pharmacist remember important things.”* (*Practicing pharmacist)*

5) *Promoting quality CMs research (2 participants)*

Two participants suggested that ‘we’ need to create a culture that encourages high quality research investigating the efficacy and safety of CMs. However, it is critical that the intellectual property and data obtained from these studies is protected to ensure an ongoing incentive for investment in such projects. The results of these studies would inform and contribute to pharmacy practice by populating infomration resources and databases. It was indicated that:

*“Unfortunately, it is the companies which can go much further in terms of really ensuring quality or really ensuring evidence, there is no incentive for them to do that. So I think that is unfortunate. As long as there is no incentive to actually do an original research, you get no advantage. If I were to fund half a million clinical trial on a particular ingredient, every other company can copy that. I think that is a failure from the regulator who is actually not promoting innovation and advance in sciences.” (Key representative of the CM industry)*

*“It goes back to the industry and research university that has the evidence for them where to from here and how do we get the government to do more research on products or how to we get the government to give some sort of IP and data protection to companies because right now I may publish a paper to support a claim and all my competitors will be using it for the next day. It is different than if you have a registered prescription product. This is some incentive we don’t see in CM.”(Key representative of a consumer advocacy group)*

6) *Collaboration among health care professionals (2 participants)*

Inter-professional communication between pharmacists and doctors about patients CMs use should be encouraged and may contribute to improving disclosure rates about CMs use. As suggested by one participant, collaborative practices like the super general practitioner clinics could see general practitioners, pharmacists, nutritionists, naturopaths and other health care providers adopt an interdisciplinary approach to patient centered care. This may encourage a collaborative view that values the consumer’s beliefs and values. It was indicated that:

*“GPs, pharmacists, nutritionists, naturopaths and all the other health care providers and the whole system should be ‘reimagined’ so there is a collaborative view with the consumer in there and their beliefs, their values and their empowerment at the center of it.”(Key representative of a consumer advocacy group)*

7) *Provision of consumer education (2 participants)*

Consumer education was also considered an important. The Australian public require a good understanding of what CMs are and the current limitations of the regulatory process. Consumer education campaigns could explain some of common or more serious interactions between CMs and standard treatments and encourage disclosure of all medicines use to doctors and pharmacists and CM practitioners. It was indicated that:

*“Consumer education is also a great issue. Australian public need better understanding of what complementary medicine is and how it is managed in Australia by regulatory processes.” (Leader within a pharmacy school)*

*“There might also be a policy to make sure people should know about the safety and efficacy of CM.” (Key representative of the CM industry)*

## Discussion

This study provides a broad yet integrative insight from the perspectives of 9 key stakeholders into the major barriers hindering pharmacists from taking up a role in CMs and their suggestions about the actions that are required to move forward. While many of the views that were shared in this study align with the findings of other studies, new and constructive opinions particularly about three main areas have emerged. These views add a valuable overview for informing and guiding quality improvement in the area of CMs in Australian pharmacy practice: CM education and training, a clearer definition of pharmacists’ responsibilities and standard of practice related to CMs, and reliable and reputable information resources.

### CM education and training

The demand for education and training in the area of CMs is a recurrent message throughout the key stakeholder interviews and is discussed in a number of other studies. [55, 65, 70, 71, 73–78]. In Australia, the quest for CM education in pharmacy training started a number of years ago [40, 63, 79]. However, the depth and breadth of CM content varies between Australian pharmacy schools. [80–82]. It has been proposed that the design of teaching about evidence-based CMs to pharmacy students should take an integrated approach [40, 83]. Specifically, focused learning about the small number of products which made up a large portion of CM sales [7, 14, 23, 77, 84, 85], analytical skills to support the pharmacists’ consultative role [38, 86, 87], communication skills to facilitate cooperation with doctors and to reduce non-disclosure [85], and ethics to optimize pharmacist’s moral sensitivity when working in a pharmacy business setting [40, 77, 87, 88] have been suggested as the building blocks of the syllabus and these suggestion were reinforced again in this study.

The importance of including CMs in CPE for practicing pharmacists has been reported elsewhere and was emphasized again in this study [84]. Factors that weigh on the decision of taking CPE on CMs were identified. Pharmacists’ personal interests, monetary cost, time and other opportunistic costs are all relevant considerations when planning CPE in CMs. The quality of evidence-based CMs CPE, the support that can help encourage pharmacists’ learning about CM, and the mandatory requirement of including CM education in individual pharmacist’s CPE plan were discussed in this study and are worth exploring further.

### Clear definition of pharmacists’ responsibility and standard of practice

In this study, there is clearly some uncertainty about the exact role pharmacists have to play related to CMs. According to the position statement issued by the Pharmaceutical Society of Australia, pharmacists assume the responsibility of providing sound evidence-based advice to assist consumers in making informed decisions regarding CMs [59]. The Australian National Medicines Policy Document defined ‘medicine’ to include prescription, non-prescription and complementary health-care products [60]. As compared to the recommendations in other studies where pharmacists were expected to counsel consumers about CMs in the same manner as they do over-the-counter medications [29, 38, 39], Australian pharmacists were expected to deliver the minimum. Nevertheless, Australian pharmacists are facing many challenges and their performance in general falls short of the basic recommendations.

At the interface of CMs and conventional medicines, pharmacists assume ethical and professional responsibilities of ensuring that consumers are properly informed about their CM use [89]. As one participant pointed out, the level of professional service and standard of practice would need to be specific to ensure that guidelines and protocols were developed within a framework of competencies. From there, knowledge and skills required by the pharmacists to handle their scope of practice could be determined. Policy-makers and pharmacy schools could then better formulate the syllabus to fulfil the training objectives which fully addresses pharmacists’ duty of care and consumer’s needs. In light of the above, it is anticipated that future studies in pharmacists’ responsibility and standard of practice would be of great relevance across a wide range of domains.

### Reliable and reputable information sources

There are 2 main issues regarding CMs information sources: lack of high quality information and lack of access to the information. The widely held opinion that there is a lack of evidence supporting the safety and efficacy of CM emerged in this study [85]. According to the participants, more quality research is needed to demonstrate the full benefits of many products in the language of evidence based medicine. However, the incentives to conduct quality clinical studies on CMs were reportedly insufficient: (1) evidence-based support was not a major influential factor for consumers’ use of CM; (2) research funding for CM was inadequate; and (3) little intellectual property protection on CM clinical studies was in place. Similar opinions have been reported previously [90], warranting further investigation.

On the reference checklist recommended by the Pharmacy Board of Australia, there are 2 evidence-based reference work on complementary and alternative medicines [91]. There were also two databases evaluated as Tier 1 sources (highest quality) as reported in a study [92]. However, only a few participants in this study could name a few of these reliable information sources. Many pharmacists were found to have difficulties in accessing the information sources and relied mainly on conventional pharmacy resources which covered only the very few CMs with clinical benefits [84, 92]. Without the knowledge of the availability of evidence based, high-quality resources, or access to it, many pharmacists (and doctors) would mistake it for a lack of evidence. It was proposed that a single, unbranded and credible information source in the form of database should be developed and made available to pharmacists with minimal access barrier. Like other studies [71, 84, 86, 93], the need to promote the availability of evidence based high-quality resources was also highlighted necessitating further studies that explore the utility of incorporating these sources in practice settings.

### Other considerations

As pointed out in this study and reported previously [94, 95], a large proportion of the counselling about CMs in the pharmacy were handled by non-pharmacist staff such as naturopaths. While naturopaths could be a source of quality CM information [22, 40, 71, 85], the current non-statuary professional registration status of naturopaths in Australia raised ethical and legal concerns for the pharmacists who heavily relied on them. Moreover, the extent of knowledge held by naturopaths regarding drug-drug interactions is likely to vary significantly between individuals due to the lack of standardization of naturopathy education and competency levels currently held in Australia. Pharmacists taking the passive stand could be easily interpreted as negligence, potentially jeopardizing the safety of the public and placing the practice of pharmacy at a reputational risk.

#### Limitations of this study

The main limitation of this study was that the findings only represented the situation in Australia. Similar studies in other countries are warranted to substantiate and compare the findings. Also, the inclusion of only one representative from most of the key stakeholder categories may not be able to provide the full capacity to reflect on the complete view about the CM-related issues relevant to the groups they are representing, warranting wider sampling in future studies. Nevertheless, the qualitative methods met the objectives of this study by providing an overview of the situation in Australia. Importantly, the findings have resulted in a proposition for specific actions to be taken that will contribute to consolidating the pharmacists’ role in CMs setting the baseline for future studies both in Australia and in other countries where there is a prevalent use of CMs.

## Conclusion

To our knowledge, this is the first interview study exploring the views of key CMs stakeholders. The relevance of the relationship between CMs and the profession of pharmacy will continue to unfold. However, the findings from this study suggest that pharmacists need to be proactive in formalizing a role that ensures the safe and appropriate use of CM products. A collaborative approach between stakeholders is required to strategically plan the implementation of this extended role that incorporates a consumer centered focus. The broad views identified in this study will contribute to formulating a model that maps the development of such a role.

## Abbreviations

CM: Complementary Medicine
CMs: Complementary medicines
CPE: Continuing professional development
OTC: Over-the-counter
TGA: Therapeutic Goods Administration
TM: Traditional Medicine
TMs: Traditional medicines
